# Near Infrared Reflectance Spectroscopy Analysis to Predict Diet Composition of a Mountain Ungulate Species

**DOI:** 10.3390/ani11051449

**Published:** 2021-05-18

**Authors:** Laia Jarque-Bascuñana, Jordi Bartolomé, Emmanuel Serrano, Johan Espunyes, Mathieu Garel, Juan Antonio Calleja Alarcón, Jorge Ramón López-Olvera, Elena Albanell

**Affiliations:** 1 Wildlife Ecology & Health Group (WE&H) and Servei d’Ecopatologia de Fauna Salvatge (SEFaS), Departament de Medicina i Cirurgia Animals, Facultat de Veterinària, Universitat Autònoma de Barcelona, Bellaterra, 08193 Barcelona, Spain; emmanuel.serrano@uab.cat (E.S.); Jordi.Lopez.Olvera@uab.cat (J.R.L.-O.); 2 Ruminant Research Group, Departament de Ciència Animal i dels Aliments, Facultat de Veterinària, Universitat Autònoma de Barcelona, Bellaterra, 08193 Barcelona, Spain; jordi.bartolome@uab.cat; 3 Wildlife Conservation Medicine Research Group (WildCoM), Departament de Medicina i Cirurgia Animals, Facultat de Veterinària, Universitat Autònoma de Barcelona, 08193 Barcelona, Spain; johan.espunyes@uab.cat; 4 Office Français de la Biodiversité, DRAS, Unité Ongulés Sauvages, Z.I. Mayencin, 38610 Gières, France; mathieu.garel@ofb.gouv.fr; 5 Departamento de Biología (Botánica), Facultad de Ciencias, Centro de investigación en Biodiversidad y Cambio Global (CIBC-UAM), Universidad Autónoma de Madrid, 28049 Madrid, Spain; juan.calleja@uam.es; 6 Centre for Research on Ecology and Forestry Applications (CREAF), 08193 Cerdanyola del Vallès, Spain

**Keywords:** diet composition, fecal NIRS, foraging ecology, global change, *Rupicapra pyrenaica pyrenaica*

## Abstract

**Simple Summary:**

Ungulates are characterized by their ability to modify or maintain habitats through their impact on plant species composition and the structure of vegetation. Assessing the diet composition of ungulates is therefore important to understand their role in the ecosystem integrity and to develop monitoring and population management plans. The diet composition of free-ranging ungulates has most often been assessed by time-consuming and cost-intensive approaches such as the direct observation of animals, chemical analysis, molecular approaches or microhistological analysis of fecal samples. Near infrared reflectance spectroscopy analysis would be a quick, economic and non-destructive alternative to assess diet composition using fecal samples. In this work, we evaluated the use of this spectroscopy method to assess the diet composition of the Pyrenean chamois (*Rupicapra pyrenaica pyrenaica*), a medium-size subalpine ungulate with a broad dietary niche. Our results support the reliability of the fecal spectroscopy analysis to monitor diet composition of free-ranging ungulates.

**Abstract:**

The diet composition of ungulates is important to understand not only their impact on vegetation, but also to understand the consequences of natural and human-driven environmental changes on the foraging behavior of these mammals. In this work, we evaluated the use of near infrared reflectance spectroscopy analysis (NIRS), a quick, economic and non-destructive method, to assess the diet composition of the Pyrenean chamois *Rupicapra pyrenaica pyrenaica*. Fecal samples (*n* = 192) were collected from two chamois populations in the French and Spanish Pyrenees. Diet composition was initially assessed by fecal cuticle microhistological analysis (CMA) and categorized into four functional groups, namely: woody, herbaceous, graminoid and Fabaceae plants. Regressions of modified partial least squares and several combinations of scattering correction and derivative treatments were tested. The results showed that models based on the second derivative processing obtained the higher determination coefficient for woody, herbaceous and graminoid plants (R^2^_CAL_, coefficient of determination in calibration, ranged from 0.86 to 0.91). The Fabaceae group, however, was predicted with lower accuracy (R^2^_CAL_ = 0.71). Even though an agreement between NIRS and CMA methods was confirmed by a Bland–Altman analysis, confidence limits of agreement differed by up to 25%. Our results support the viability of fecal NIRS analysis to study spatial and temporal variations of the Pyrenean chamois’ diets in summer and winter when differences in the consumption of woody and annual plants are the greatest. This new use for the NIRS technique would be useful to assess the consequences of global change on the feeding behavior of this mountain ungulate and also in other ungulate counterparts.

## 1. Introduction

Information about plant species composition and the quality of animal diets is important for wildlife researchers in order to monitor the animal nutritional condition and to predict population dynamics. Knowledge of the range of herbivore diet quality is essential for a sustainable management of natural resources. Vegetal landscapes are fast-changing due to global warming and land-use transformation [1,2,3] which affect the quality and availability of forages and probably the foraging behavior of ungulates [4,5]. Therefore, detecting diet change trends may be useful for uncovering clues about how climate, landscape and vegetation changes affect the diet of free-ranging herbivores, their health condition and their chances of overcoming climate change.

A wide variety of invasive and non-invasive methods have been used to assess the diet composition of wild ungulates (for further information, see Appendix A). Invasive methods are inappropriate for continuous monitoring over time, especially for protected populations or out of the hunting season, since animal capture [6] or shooting [7,8] is required. Non-invasive methods, however, allow an assessment of diet composition without animal behavior interference. Traditionally, researchers have relied on fecal cuticle microhistological analysis (CMA) of herbivore feces to determine diet composition through the study of plant cuticle [9,10] since it is the cheapest diet estimation method compared to the other non-invasive methods. This indirect assessment has been by far the most common technique for assessing diet selection in both domestic [11] and wild ruminants [12,13,14]. In spite of its advantages, the histological identification of plant fragments in feces is a very laborious and time consuming task, and presents other disadvantages which were discussed by Holechek et al. [9]. For these reasons, a reliable, quick and cheap non-invasive predictor method is needed for diet composition studies.

Near infrared reflectance spectroscopy (NIRS) is a non-destructive, emission-free and low-cost predictive technique which needs low amounts of samples and reduces the use of conventional time-consuming and laborious methods. The NIRS technique has already been used to predict nutritional parameters and diet quality in fecal samples (fecal NIRS) of domestic (e.g., sheep [15], cattle [16]) and wild animals (e.g., Pyrenean chamois, *Rupicapra pyrenaica pyrenaica* [17]; red deer, *Cervus elaphus* and roe deer, *Capreolus capreolus* [18]; white-tailed deer, *Odocoileus virginianus* [19]) and other herbivores species [15,20,21,22,23,24]. Some studies used fecal NIRS to determine the plant species consumed by domestic animals (cattle [25], sheep [26,27], cattle and sheep [28] and pigs [29]). However, few works have been carried out to achieve a NIRS prediction model for the diet composition of wild ungulates, most likely because the broad dietary niche of these animals is a challenge for NIRS calibration. Nevertheless, in a previous NIRS study, we applied it with success, using a multispecies calibration model obtained from fecal samples of four wild herbivores [30].

The European alpine grasslands are threatened due to a process of shrubification that is linked to land abandonment and climate change [31,32,33]. Thus, grazing pressure may be exacerbating the threat in the remaining open areas and disrupting the herbivore–plant relationships at different spatial and temporal scales. A cheap, quick and reliable diet composition assessment technique would provide valuable information to monitor the nutritional condition of wild mountain herbivores, to find out how climate warming and land use shifts are affecting these animals and to predict their population dynamics.

The aim of our study was to explore the pros and cons of fecal NIRS to predict the diet composition of mountain ungulates, using the fecal CMA as the reference method, since, as mentioned above, it is the most common technique for diet composition assessment. We focused on the Pyrenean chamois, a medium-sized mountain-dwelling ungulate widely distributed in subalpine and alpine habitats of the Pyrenees [34].

## 2. Materials and Methods

### 2.1. Sampling Area

**Figure 1:**
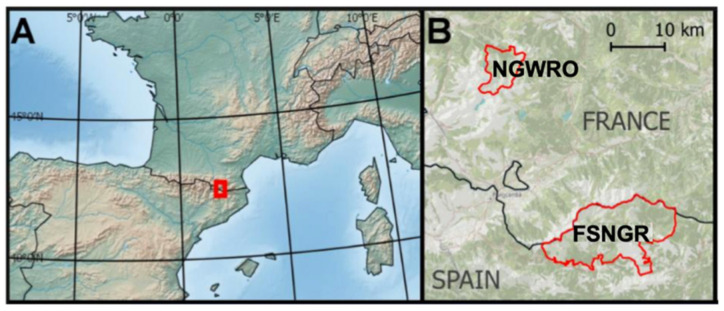
The two study areas. (**A**) Map of south-western Europe with the location of our study areas noted with a red rectangle. (**B**) Zoom in on the previous red rectangle with the location of the National Game and Wildlife Reserve of Orlu (NGWRO) in France and the Freser-Setcases National Game Reserve (FSNGR) in Spain (both delimitated by a red line). Thick black line represents France–Spain border.

Chamois fecal samples were collected in the Freser-Setcases National Game Reserve (FSNGR) and the National Game and Wildlife Reserve of Orlu (NGWRO, Figure 1). The FSNGR is located on the southern side of the eastern Pyrenees, Spain (42°22′ N, 2°09′ E, see Figure 1). This is a mountainous area of 20,200 ha with an altitude average of 2000 m above sea level (m.a.s.l.) ranging from 1200 to 2910 m.a.s.l. The annual mean temperature is 6.0 °C and the mean yearly accumulated rainfall is 963.4 mm (data from Núria meteorological station located at 1971 m.a.s.l. in the core FSNGR, Servei Meteorològic de Catalunya (www.meteocat.com accessed on 29 October 2014). The FSNGR vegetation is represented by sub-Mediterranean, medio-European and boreo-alpine vegetation communities linked to acidic and calcareous soils of the eastern Pyrenees [35,36]. At lower altitudes, the vegetation is dominated by oak (*Quercus* spp.) and Scots pine (*Pinus sylvestris)* forests with anthropic grasslands. At higher altitudes, the landscape is a mosaic of patches of mountain pine (e.g., *Pinus uncinata*), shrublands (*Cytisus oromediterraneus* and *Juniperus communis* subsp. *alpina*) and subalpine grasslands (*Festuca* spp., *Nardus stricta*, *Trifolium alpinum*).

The NGWRO is also located in the eastern Pyrenees, but on the northern side, in France (42.66° N, 1.97° E, see Figure 1). It is a protected area of 4250 ha ranging from 920 to 2765 m.a.s.l. The area presents a mountainous oceanic climate with annual mean temperatures of 8 °C and a mean yearly accumulated rainfall of 1200 mm (meteorological data collected by Meteo France weather station of Ascou-Pailhères, 1120 m.a.s.l., 42.72° N, 1.89° E). The vegetation is mainly composed of beech forests (*Fagus sylvatica*) with Atlantic chamaephytes and hemicriptophytes in the montane stage. In contrast, the subalpine stage is dominated by mosaics of shrublands of *Rhododendrum ferrugineum* and *Vaccinium* spp., grasslands of *Festuca* spp. and *Nardus stricta* and mountain pine (*Pinus uncinata*) patches. In the higher slopes, grasslands, rocks and cliffs are the prevailing habitats [37].

### 2.2. Fecal Sampling Procedure

In the FSNGR, 449 fresh chamois fecal samples were collected from May 2009 to May 2012. Each month, the study area was surveyed by two observers following defined transects of about 5 km each. Covering the whole altitude range and the main vegetation communities of the study area, the observers located chamois groups using 10 × 42 binoculars and 20 − 60 × 65 spotting scopes. Once the group size, composition and precise location of the chamois were recorded, observers collected fresh droppings at the exact place where animals were sighted and their surroundings. In the NGWRO, 361 fecal samples were collected from April 1992 to May 2015 in a long-term capture–mark–recapture monitoring project. Most captures in NGWRO occurred in spring (April-May). The number of animals trapped each year varied from 2 to 66, using different methods such as corral nets, drive nets, snares, or tele-anesthesia. In both study areas, fecal samples were placed in individually labelled plastic bags and transported to the laboratory where they were frozen at −20 °C for further laboratory analysis.

### 2.3. *Fecal Cuticle Microhistological Analysis*

From all the collected samples, 192 randomly chosen samples were microhistologically analyzed for this study (95 from FSNGR and 97 from NGWRO). The procedure employed was developed based on work by Stewart [38]. Once samples were thawed, part of each sample was water-washed to remove extraneous material and then ground in a mortar to separate the epidermal fragments. After that, 10 g of sample were placed in a test tube with 5 mL of 65% concentrated HNO_3_. The test tubes were then boiled in a water bath for 1 min. After digestion in HNO_3_, the samples were diluted with 200 mL of water. This suspension was then passed through 1.00 and 0.25 mm filters. The 0.25–1.00 mm fraction was spread on glass microscope slides in a 50% aqueous glycerin solution and cover-slips were fixed with DPX microhistological varnish. Two slides were prepared from each sample. Later, the slides were examined by the same operator under a microscope at × 100 and × 400 magnifications and plant fragments were recorded and counted until 200 leaf epidermis units were identified. An epidermis collection of 55 main plant taxa of the study area was made and used as a reference method for fragment identification [39]. Plant taxa were pooled into two main functional groups, namely: woody species and herbaceous species. The group of woody species was composed by the genera *Calluna*, *Cytisus*, *Genista*, *Hedera*, *Juniperus*, *Pinus*, *Quercus*, *Rosa*, *Rhododendron*, *Rosmarinus*, *Rubus, Teucrium, Thymus* and *Vaccinium*. The group of herbaceous species was composed by *Anthyllis, Arrhenatherum, Astragalus, Avenula, Carex, Festuca, Juncus, Lotus, Nardus, Poa, Trifollium*, and 14 more genera. Moreover, regardless of this dichotomous classification, the Fabaceae group was also considered as well as graminoid species, which includes Poaceae, Juncaceae and Cyperaceae families. This CMA was considered as the gold standard method for our NIRS calibration.

### 2.4. NIRS Analysis and Spectral Data Analysis

To perform the NIRS analysis, the material not used in the CMA was dried at 60 °C in a stove for 48 h. After, it was milled in a Cyclotec mill of 0.5 mm screen (FOSS Tecator, Höganäs, Sweden) and packed in a ring-cup sample cell. The samples were then scanned from 1100 to 2500 nm using a NIRSystems 5000 scanning monochromator (FOSS, Hillerød, Denmark) using a PbS detector. The analysis was carried out in duplicate using a closed ring cup cell (35 mm diameter) with quartz glass windows containing 2–3 g of the sample and absorbance was recorded as log (1/reflectance) (log 1/R) at 2 nm intervals, resulting in 692 data points for each sample. All measures were performed by the same operator.

One hundred and fifty samples were used for calibration (approximately 78% of the total samples) and 42 random samples (around 22% of the total samples) were separated previously and used as an external source of samples for validation. A WinISI III (v1.6; Infrasoft International, Port Matilda, PA, USA) software program was employed for spectra data analysis to develop chemometric models. The spectra were corrected emphasizing peaks and valleys and reducing noise for the effects of light scattering and particle size, using the standard normal variate, detrend (DT) or multiplicative scatter correction (MSC). Several prediction models were tested, taking into account different independent factors in addition to major group plants (woody/herbaceous, graminoids and Fabaceae), such as the study area (NGWRO/FSNGR) and the season. The prediction models were performed by modified partial least square regression using first and second derivatives of the spectra. In short, the results of the calibration were checked by observing (*t*) and global (GH) outliers, with the extreme values of *t* > 2.5 and GH > 10 not being considered for calibration.

The optimum prediction model was selected on the basis of the minimum standard error of calibration (SEC) and greatest coefficient of determination (R^2^_CAL_). Performance of prediction model was evaluated using: (i) the coefficient of determination of the external validation (R^2^_VAL_); (ii) the ratio of performance to deviation (RPD), defined as the ratio of the range in the reference data from the validation set to the standard error of prediction (SEP); and (iii), the range error ratio (RER), described as the ratio of the range in the reference data (validation set) to the SEP. Additionally, we also considered other statistics, such as bias to measure the accuracy between predicted (by NIRS) and reference values (MCA) in an external validation. For a detailed description, see [40,41].

### 2.5. Relationships between Fecal CMA and NIRS Methods

We explored the relationships between fecal CMA and NIRS by two approaches: model selection and Bland–Altman regressions in order to test agreement between methods. The model selection approach was used to explore whether the relationships between diet composition predicted by NIRS and diet composition estimated by the CMA varied between plant groups. A model selection procedure based on the Akaike information criterion (AIC) was performed [42]. The model with the lowest AIC was retained, and the remaining competing models were ordered according to their Akaike differences (Δi) with respect to the best model (lowest AIC). The Akaike weight (Wi) for each competing model was also calculated. Later, we used the Bland–Altman analysis to explore the agreement between the proportion of each functional group of plants assessed by the CMA and the predictions made by our NIRS analysis [43]. This graphical method is the most popular for comparing two measurement techniques by means of the representation of the differences (Y-axis) and magnitude of such measurements (X-axis, for a review of agreement approaches, see Zaki et al. [44]). The limits of agreement (LoA) at 95% defined as the mean differences ± 1.96 × SD (standard deviation), are also estimated and represented. Bland–Altman analysis was performed in the “blandr” package version 0.5.1 [45], the R software 4.0.2 version [46].

## 3. Results and Discussion

### 3.1. Spectral Characteristics of Samples

**Figure 2:**
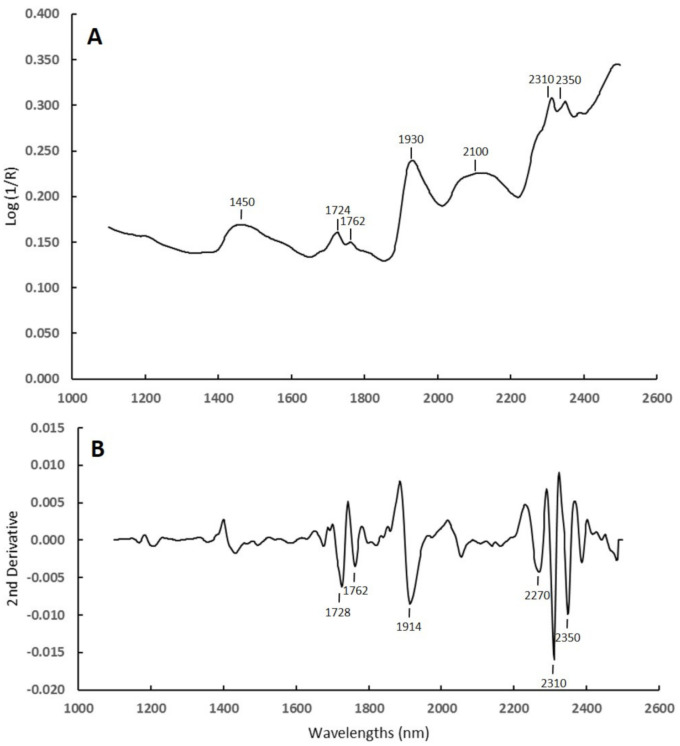
Near infrared reflectance spectra used to build a prediction model for diet composition of Pyrenean chamois. It shows the bands of the main absorption. (**A**) Raw average spectrum of fecal samples, (**B**) the same spectrum after a second derivative and detrend treatment (R = reflectance).

Figure 2A shows the average raw NIR spectrum of fecal samples used. Regardless of their plant species composition, all fecal samples showed local peaks at 1450, 1724, 1762, 1930, 2100, 2310 and 2350 nm. These absorption peaks were similar to those reported previously by Villamuelas et al. [22] in Pyrenean chamois fecal samples, where NIRS was able to predict fecal nitrogen using a multispecies calibration. A derivative transformation was performed on the raw spectra to narrow the bandwidths and also remove some of the baseline variations. This transformation facilitated the analysis of the results. It made the absorption bands much more evident since overlapping absorbances become separated and the peak resolution was improved. Figure 2B shows the average second derivative NIR spectrum of fecal samples analyzed. The same trend of absorption bands of major components as in the raw spectra were observed in this derivative transformation. However, the absorption bands at 1914 and 2270 nm became clearer than in the raw spectra. Shenk et al. [47] reported that the peak at 1914 nm is associated with the absorption of the OH stretch while the 2270 nm wavelength is due to overtone and combination CH stretches from the various CH group absorption bands, probably related to water and lignin content, respectively. Cozzolino et al. [48] found similar derivative spectra in beef fecal samples.

### 3.2. Development of Prediction Models

Descriptive statistics of both calibration and validation sets are shown in Table 1. The broad ranges observed in the values were probably due to the sampling method, which covered large sampling zones, located in two different areas, over several years through two contrasted seasons, and showed the wide composition variability of the chamois diet. The Pyrenean chamois has already been described as an intermediate feeder, capable of adapting its diet composition to woody or herbaceous plants depending on their availability [49,50]. A high range in the reference values, allows a better establishment of prediction through NIRS. Furthermore, the calibration and validation matrices covered a similar broad range, thus ensuring the robustness of the calibration models [51].

Table 2 shows the statistical results from NIRS calibration equations and their corresponding validations (both cross and external validations). The best math treatment for each category was selected from those with lower SECV and/or SEC values. The best prediction model results were obtained from second derivative combined with MSC, DT or without scatter correction. Other prediction models were tested taking into account other independent factors than the major plant groups such as study area (NGWRO/FSNGR) or season (winter/summer), but neither of them showed better prediction models.

We achieved acceptable predictive models for woody, herbaceous and graminoids composition (Table 2), since NIRS equations owning a coefficient of determination in calibration (R^2^_CAL_) ≥ 0.80 are considered acceptable [40,52]. The SEC and SECV values were also acceptable for woody and herbaceous, since for a good predictive power, SECV value must be close to SEC [40,52]. Likewise, RPD values for woody and herbaceous suggested prediction models appropriate for screening purposes (between 2.0 and 2.4) according to some studies [40,41,53]. However, lower RPD values were found for graminoids and Fabaceae plants (1.79 and 1.47, respectively), which indicated a poor prediction model (RPD ≤ 1.9) [40,41,53]. Those low RPD values in graminoids and Fabaceae plants might be partially explained due to the fact that these two groups are more taxa specific and their predictions are not so accurate as generalist ones (woody and herbaceous). In addition, woody plants are easier to detect in fecal analysis because they are less digestible than the other groups [54,55,56] and lignin is easily detectable in NIR analysis [48].

Other works provided similar or better results developing fecal NIRS prediction models to estimate plant species diet composition in livestock: cattle (R^2^_CAL_ = 0.94 just for monocots and dicots proportions [21,25]); goat (R^2^_CAL_ = 0.95 to 0.99 depending on the plant taxa hay, *Pistacia lentiscus, Phillyrea latifolia* and *Pinus brutia* [57]; R^2^ = 0.85 for herbaceous vegetation as one category; R^2^ = 0.89 for *Phillyrea latifolia*; R^2^ = 0.77 for tannin-rich *Pistacia lentiscus* [58]); and sheep (R^2^_CAL_ = 0.96 for *Artemisia tridentata* [59]; R^2^_CAL_ = 0.86 to 0.97 depending on the plant taxa species: alfalfa, cereal straw, maize and forage [27]). The better NIRS prediction power in plant taxa diet composition in the livestock studies may be due to the fact that the reference method was the actual diet intake, since diets were already known and very few species were represented in those diets. However, in these studies, researchers used the direct observation of bites [58] or controlled formulation diets [21,25,26,27,28] as reference methods, which are not viable approaches for wild herbivore studies as in the case of the CMA.

To the best of our knowledge, only two studies have used microhistological identification as a reference method in wild ruminants. Our previous work [30] for a multispecies calibration obtained from fecal samples of four wild herbivores (red deer, Barbary sheep, *Ammotragus lervia,* mouflon, *Ovis orientalis musimon* and rabbit, *Oryctolagus cuniculus*) reached values of R^2^_CAL_ = 0.98 and R^2^_CAL_ = 0.97 for woody and herbaceous components, respectively. Jean et al. [60] were also able to predict the amount of coniferous fragments in the diet of wild ruminants (white-tailed deer) with a similar precision to our results (R^2^_CAL_ = 0.89).

**Figure 3:**
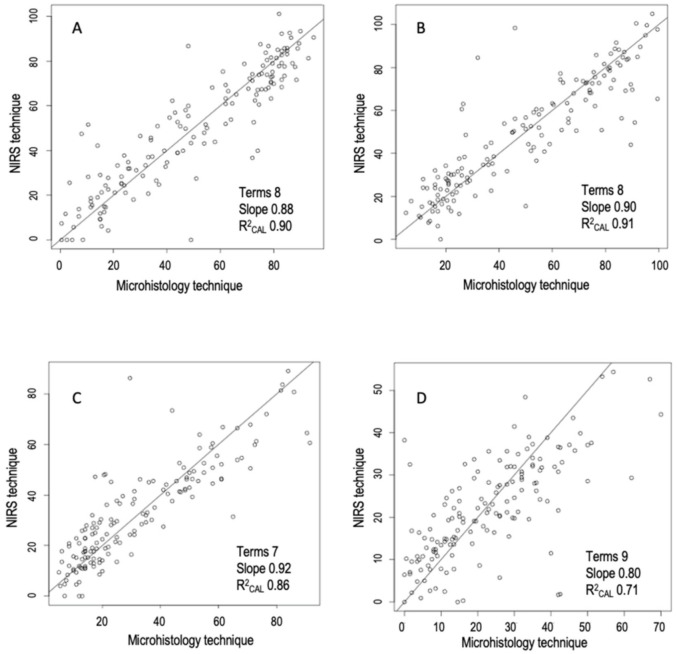
Linear regression between fecal cuticle microhistological analysis and near infrared reflectance spectroscopy predictions for four functional groups of plants: woody (**A**), herbaceous (**B**), graminoids (**C**) and Fabaceae (**D**) found in 150 fecal samples of Pyrenean chamois. The numbers of terms, the slope and the coefficient of determination for calibration (R^2^_CAL_) are also shown.

In Figure 3, the best regressions obtained between NIRS prediction and reference data for woody (A), herbaceous (B), graminoids (C), and Fabaceae (D) are represented. Good models should result in a slope very close to 1.0, and this was the case for woody, herbaceous and graminoids. The number of PLS factors or terms (Figure 3) is a measure of the potential effectiveness of the calibration, which, according to Williams et al. [61], should range from six to eight factors.

### 3.3. Comparison between NIRS Predictions and Microhistologic Method

**Figure 4:**
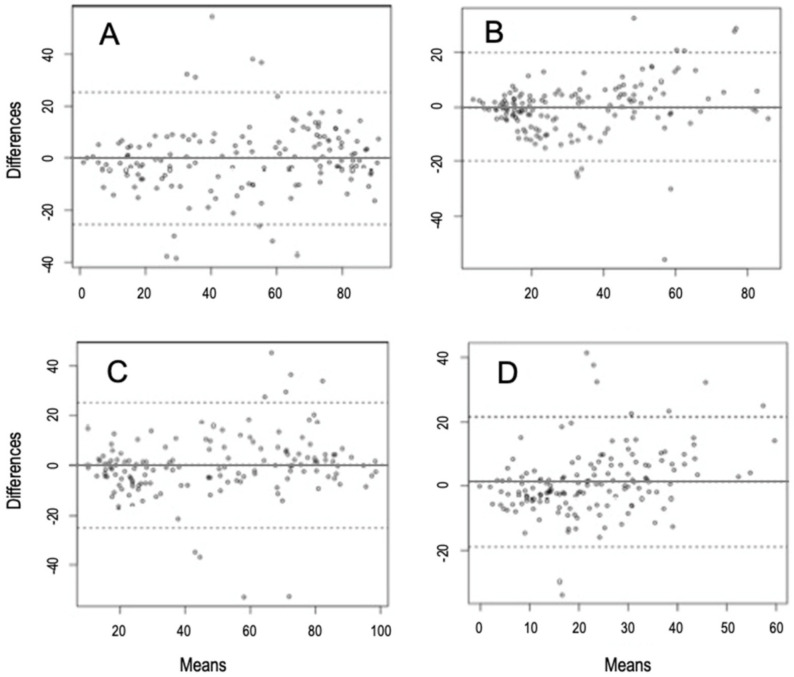
Bland–Altman plots showing the agreement between fecal cuticle microhistological analysis (CMA) and near infrared reflectance spectroscopy (NIRS) predictions for four functional groups of plants: woody (**A**), herbaceous (**B**), graminoids (**C**) and Fabaceae (**D**) found in fecal samples of Pyrenean chamois. Y axis represents the difference between the proportion of each functional group of plants observed by the CMA and the NIRS predictions. X axis represents the mean of the observed and the predicted proportion for each functional group of plants. The limits of agreement (dotted line), from-1.96SD (standard deviation), to +1.96SD have also been represented.

The best model to explain the observed variability of CMA and NIRS methods for predicting diet composition is independent of the plant group analyzed (ω_i_ = 0.85, Table 3). Therefore, NIRS method predictions and MCA estimates did not vary across plant groups. In the same line, the Bland–Altman analysis showed a good agreement between NIRS and MCA for all functional groups of plants (Table 4). In this analysis, the bias indicates the average differences between the CMA and the NIRS predictions for each functional group recorded in our fecal samples. Proximity to zero indicates a good agreement in the detection of plants by both methods. Both bias and the number of observations outside the upper and lower limits of agreement were lower for woody, herbaceous and graminoid plants, with Fabaceae species showing higher values for both indicators. Altogether, this agrees with MCA and NIRS showing a better correspondence for the first three groups of plants than for Fabaceae. However, in spite of these differences, the lower and higher confidence limits of agreement were overall similar for each plant group (Table 4 and Figure 4). Overall, this analysis confirmed that both MCA and NIRS methods are interchangeable to predict the fecal diet composition in Pyrenean chamois for the groups of plants studied.

Our study showed that NIR spectra of feces can be used to predict major groups of plants (namely woody and herbaceous, and even for graminoids) as a diet composition proxy for the Pyrenean chamois. Regarding the better results in livestock studies with known diet components as a reference method, it could be suggested that the CMA may not be the ideal reference method. Still, it is currently the best available noninvasive approach to study the diet of wild and protected species. The main disadvantage of the CMA is that highly digestive plant species could be underrepresented in the estimated diets, whereas less digestible plants could be overrepresented [62]. Shaffer et al. [63] and Volesky et al. [28] achieved better results estimating plant species composition using NIRS (R^2^_CAL_ = 0.96 for both) with samples collected directly from the field or from domestic animals esophageal extrusa, respectively. Those better results could be adjudged to the fact that field and esophageal extrusa samples were not digested or not totally digested. Several authors [9,64,65,66] pointed out that the differing degrees of digestibility of plant species must be taken into account, as long as it may determine fragment detection in fecal samples. Espunyes et al. [67] proposed an index of preservation for each plant species to link the relative quantity of a specie detected in fecal sample to the corresponding ingested quantity. Plant digestive pattern is not the only problem with this method; CMA requires a good knowledge of the micromorphology of different plant tissues, and human error, if untrained, could be quite important when identifying some plant groups.

Our current prediction models show some limitations in accurately depicting the diet composition of the Pyrenean chamois, probably due to digestibility differences between woody and herbaceous plants, leading to an overestimation of the woody plants portion in diet composition. However, we show that our models can be used as a screening technique for woody and herbaceous taxa, particularly when or where chamois diet woody/herbaceous ratio is highly contrasted (e.g., summer/winter) [50]. In the context of alpine ecosystem, the Pyrenean chamois’ diet shows a relative abundance of woody species, improving, with that feature, the screening monitoring. The reason that NIRS could be used as a rough diet composition proxy is probably due to high woody species percentage (more than 70%) and that subalpine and alpine ecosystems are relatively simple, with only a few winter forage types available to intermediate feeders.

This is a relatively new application for NIR spectra, since previous research on wild ungulates primarily focused on estimates of the nutritional quality of diet rather than its plant species/groups composition [18,19]. Although we expected to predict the four forage groups, being able to predict just woody species and herbaceous content is already quite useful for diet composition and ecological studies. Alpine ecosystems are changing in a very fast way due to changes in land uses and global warming. Therefore, better tools to evaluate the impact of such environmental changes on animal–plant interaction are needed. Taking into account that feeding habits are crucial to population dynamics, the NIRS method to predict plant taxa in the Pyrenean chamois’ diet composition may provide important information about diet quality and possible changes in vegetation availability.

## 4. Conclusions

Our results demonstrated the potential of fecal NIRS to estimate major plant groups composition for the Pyrenean chamois as a quick, cheap and practical option, with good accuracy but limited precision power. Even though fecal samples do not provide the actual diet composition but an estimate, this information is adequate enough for rough analysis, for monitoring purposes (e.g., to compare independent variables effects on diet preference) focused on major plants groups. The NIRS method, however, would not be enough if it is necessary to know the actual composition of the diet with high precision and/or at genus and specie level.

## Figures and Tables

**Table 1:** Plant species composition (%) of Pyrenean chamois fecal samples used in the calibration and validation sets.

	Calibration Set				Validation Set			
	N	Range	Mean	SD	n	Range	Mean	SD
Woody	150	0.5–95.0	50.42	28.17	42	3.5–87.5	46.26	26.83
Herbaceous	150	5.0–99.5	48.90	27.68	42	12.5–96.5	53.11	26.55
Graminoids	150	5.0–91.5	32.04	21.47	42	10.0–75.0	33.39	19.71
Fabaceae	150	0.0–70.0	22.69	15.16	42	1.5–55.0	22.69	13.49

Number of samples for calibration (N), number of samples for validation (n), interval between the maximum and the minimum value of data set (range), standard deviation (SD).

**Table 2:** Calibration and validation statistics of prediction models used to determine the diet composition (% presence) in Pyrenean chamois fecal samples by near infrared reflectance spectroscopy analysis.

	Calibration				Cross Validation		External Validation					
	Math Treatment ^a^	Scatter ^b^ Correction	R^2^_CAL_	SEC	R^2^_cv_	SECV	R^2^_VAL_	SEP	Bias	Slope	RPD	RER
Woody	2,5,5,1	MSC	0.90	9.39	0.85	11.10	0.83	11.29	−0.94	0.88	2.38	7.44
Herbaceous	2,4,4,1	none	0.91	8.49	0.82	11.48	0.81	11.88	2.82	0.90	2.24	7.07
Graminoids	2,4,4,1	DT	0.86	7.70	0.71	11.24	0.70	11.03	0.74	0.92	1.79	5.89
Fabaceae	1,4,4,1	none	0.71	7.81	0.52	9.79	0.55	9.20	1.39	0.80	1.47	5.82

^a^ Math treatment: derivative order, subtraction gap, first smoothing, second smoothing. ^b^ MSC multiple scatter correction, DT detrend. R^2^_CAL_ coefficient of determination for calibration, SEC standard error of calibration, R^2^_cv_ coefficient of determination for cross validation, SECV standard error of cross validation, R^2^_VAL_ coefficient of determination for external validation, SEP standard error of prediction, RPD ratio of performance to deviation (SD/SEP), RER range error ratio (range/SEP).

**Table 3:** Model selection to explore whether the relationships between diet composition of Pyrenean chamois (% presence) assessed by the cuticle microhistological method (reference method) and predicted by near infrared reflectance spectroscopy varies among woody, herbaceous, graminoids and Fabaceae groups.

Selected Model	K	AIC	Δi	ω_i_
**NIRS method**	**3**	**4616.2**	**0.00**	**0.852**
NIRS method + Group plant	6	4619.7	3.49	0.148
NIRS method * Group plant	9	4625.0	10.08	0.005
Group plant	5	5420.7	804.93	0.000

K = number of parameters, Δi = difference of AIC with respect to the best model, Wi = Akaike weight. The best model is indicated in bold.

**Table 4:** Descriptive statistics for the Bland–Altman analysis of agreement between the fecal cuticle microhistological analysis and near infrared reflectance spectroscopy predictions for four functional groups of plants found in fecal samples of Pyrenean chamois, collected in the French and Spanish Pyrenees.

Functional Group	Parameter	Mean Value	CI at 95%	
			Minimum	Maximum
Woody	Mean differences (bias)	−0.05	−2.1	2.04
	ULoA	25.46	21.87	29.07
	LLoA	−25.57	−29.17	−21.97
Herbaceous	Mean differences (bias)	0.14	−1.91	2.21
	ULoA	25.18	21.65	28.71
	LLoA	−25.01	−28.42	−21.36
Graminoids	Mean differences (bias)	−0.05	−1.69	1.58
	ULoA	19.87	17.17	22.79
	LLoA	−19.98	−17.17	−22.79
Fabaceae	Mean differences (bias)	1.28	−0.37	2.94
	ULoA	21.46	18.61	24.31
	LLoA	−18.88	−21.721	−16.04

Confidence interval (CI), standard deviation (SD), upper and lower limits of agreement (ULoA and LLoA, respectively).

## Data Availability

The data presented in this study are available on request from the corresponding authors. The data are not publicly available due to project IP rules.

## Appendix A

**Table A1:** Systematic review on the use of some diet composition determination methods for both, domestic and wild ruminants. In the column ‘Pros’ correspond to the advantages and in the column ‘Cons’ correspond to the disadvantages for each method.

Type	Technique		Pros	Cons	Authors
Invasive methods	Rumen content		- Direct sample observation - Good estimation	- Inappropriate for continuous monitoring over time and/or protected species - Some species may be finely masticated and/or highly digested through digestive tract	[68]
	Esophageal fistula				[69]
Non-invasive methods	Direct grazing animal observation		- Simple - Small material investment - Can determine the species and plant parts that are consumed	- The accuracy strongly depends on the degree of training of the observer - Time consuming	[70,71]
	Video recording				[72]
	Fecal analysis	Cuticle microhistological analysis	- No animal stress infringed - Small material investment	- No quantitative method - Some species are highly digested through digestive tract - Time consuming - Long specialist training period - The accuracy strongly depends on the degree of training of the specialist	[9,10,11,12,13,14]
		n-alkane markers (wax components)	- Useful on simple dietary mixtures of up to four components (livestock)	- Not effective enough on complex diets (wild herbivores) - Expensive - Time consuming	[73,74]
		Isotopes			[75]
		DNA-barcoding	- The most powerful diet assessing method	- Variations in DNA content and different digestibility of several plants limit its accuracy - Long previous work period - Highly expensive	[76]
